# Detection of *bla*_KPC_ and *bla*_NDM_ carbapenemase genes among *Klebsiella pneumoniae* isolates in Addis Ababa, Ethiopia: Dominance of *bla*_NDM_

**DOI:** 10.1371/journal.pone.0267657

**Published:** 2022-04-27

**Authors:** Tewachew Awoke, Brhanu Teka, Abraham Aseffa, Shemse Sebre, Aminu Seman, Biruk Yeshitela, Tamrat Abebe, Adane Mihret

**Affiliations:** 1 Department of Medical Laboratory Sciences, College of Medicine and Health Sciences, Bahir Dar University, Bahir Dar, Ethiopia; 2 Department of Microbiology, Immunology, and Parasitology, School of Medicine, College of Health Sciences, Addis Ababa University, Addis Ababa, Ethiopia; 3 Armauer Hansen Research Institute, Addis Ababa, Ethiopia; Ross University School of Veterinary Medicine, SAINT KITTS AND NEVIS

## Abstract

**Background:**

Infections caused by *Klebsiella pneumoniae* have been difficult to control because of the worldwide emergence of carbapenem-resistant isolates mainly due to carbapenemase production. Information regarding carbapenemase-producing *K*. *pneumoniae* is still scarce in Ethiopia. Therefore, the current study aimed to determine the prevalence of carbapenemase-producing *K*. *pneumoniae* and to assess the occurrence of *bla*_NDM_ and *bla*_KPC_ carbapenemase genes_._

**Methods:**

A cross-sectional study was conducted from September 2018 to February 2019 at Tikur Anbessa Specialized Hospital, Addis Ababa, Ethiopia. A total of 132 non-duplicate *K*. *pneumoniae* isolates were studied. Phenotypic confirmation of carbapenemase production was done by modified Carbapenem Inactivation Method (mCIM). Multiplex PCR was performed for the detection of carbapenemase-encoding genes *bla*_KPC_, and *bla*_NDM_.

**Results:**

Out of the total 132 *K*. *pneumoniae* isolates, 39 (29.6%) were non-susceptible to one or more carbapenems. The prevalence of carbapenemase-producing isolates from the total was 28 (21.2%) with mCIM of which the most dominant gene was *bla*_NDM_ 26 (92.9%) and one isolate carried *bla*_KPC_ concomitantly. Carbapenemase-producing *K*. *pneumoniae* isolates were 100% non-susceptible to half of the antimicrobials used in the study, including meropenem and ertapenem. Previous use of carbapenems was associated with carbapenemase production (P = 0.004).

**Conclusions:**

The prevalence of carbapenemase-producing *K*. *pneumoniae* isolates was worrying in the study area. To our knowledge, the study described the emergence of *bla*_NDM_ and *bla*_KPC_ gene carrying *K*. *pneumoniae* in Ethiopia for the first time. Further large-scale molecular-based studies, including other carbapenemase genes and sequencing of *K*. *pneumoniae*, are warranted to have a clear awareness about the presence of antimicrobial resistance high-risk clones in Ethiopia.

## Introduction

Carbapenems have a carbapenem together with the beta-lactam ring which makes them more stable against most β-lactamases [1]. They are the most effective against Gram-positive and Gram-negative bacteria. According to the Clinical and Laboratory Standards Institute (CLSI) guidelines meropenem, imipenem, ertapenem, and doripenem are recommended treatments for infections caused by *Enterobacteriaceae* [2]. Their effectiveness, stability, and fewer adverse effects compared to other last-line drugs such as polymyxins make them the most reliable last-resort treatments for bacterial infections [1]. The prevalence of ESBL-producing *K*. *pneumoniae* such as CTX-M-15-producers continues to impose a serious threat to human health [3], and carbapenems are widely considered as the drugs of choice for the treatment of severe infections caused by Extended-spectrum β-lactamases (ESBL)-producing *Enterobacteriaceae* [4]. However, in recent years, carbapenem-resistant *Enterobacteriaceae* particularly *K*. *pneumoniae* is rising alarmingly [5, 6].

Resistance to carbapenems is mainly through carbapenemase enzyme production. Productions of other enzymes that have weak carbapenemase activity such as ESBLs and AmpC β-lactamases together with porin alteration, drug efflux pumps, as well as alterations in penicillin-binding proteins are also mentioned as additional resistance mechanisms [1, 5–7]. Based on their molecular structures (Ambler classification system) carbapenemases belong to class A, B, and D of β-lactamases [5–8].

*K*. *pneumoniae* has caused hospital outbreaks in different countries [9], requiring early detection of carbapenemases in infected patients and/or carriers to prevent the occurrence of outbreaks. It has been indicated that house flies are potential vectors of antibiotic-resistant *K*. *pneumoniae* [10]. *Klebsiella pneumoniae* carbapenemases (KPCs) are the most common transmissible class A carbapenemase circulating in *Enterobacteriaceae* predominantly in *K*. *pneumoniae* worldwide mainly due to clonal expansion of strains of *K*. *pneumoniae* [8]. Unlike KPC the rapid and dramatic dissemination of New Delhi metallo-ß- lactamase (NDM)-producing *Enterobacteriaceae* is mediated by promiscuous plasmid not associated with dominant clonal strains [5].

Three *bla*_NDM-1-_positive *Acinetobacter baumannii* isolates were reported from Jimma, Ethiopia [11]. However, to the best of our knowledge, no *bla*_NDM_- and *bla*_KPC_-carrying *K*. *pneumoniae* have been described in Ethiopia so far. Therefore, the current study aimed to determine the prevalence of carbapenemase-producing *K*. *pneumoniae* and to assess the occurrence of *bla*_NDM_ and *bla*_KPC_ carbapenemase genes.

## Materials and methods

### Study population

A total of 132 study participants who were visited Tikur Anbessa Specialized Hospital (TASH), Addis Ababa, Ethiopia and became culture-positive for *K*. *pneumoniae* over six months (from September 2018 to February 2019) were enrolled conveniently. Preliminary identification of *K*. *pneumoniae* was done by inoculating the specimens on MacConkey agar (Oxoid, UK) and 5% sheep blood agar (Oxoid, UK). Further identification was done through Gram stain, and a series of biochemical tests including indole, triple sugar iron agar, citrate utilization, mannitol, malonate, lysine decarboxylase, urea agar, and motility medium. *K*. *pneumoniae* is Gram-negative and rod-shaped, lactose fermenter, indole negative, gas and acid producer, hydrogen sulfide negative, citrate positive, mannitol fermenter, malonate positive, lysine decarboxylase positive, urea slow producer, and non-motile [12, 13]. Socio-demographic characteristics and clinical information of the study participants were obtained using a well-designed questionnaire and from their medical records by health care workers.

### Antimicrobial susceptibility testing

Using a sterile wire loop, 3–5 single colonies were picked from blood agar and emulsified in 3–4 ml normal saline to prepare a 0.5 McFarland standard using McFarland Densitometer. From the standard, cells were spread onto Muller-Hinton agar (Oxoid, UK) using a sterile swab for the Antimicrobial Susceptibility Testing (AST) [12]. The AST was performed based on the Kirby–Bauer disc diffusion method using the following antimicrobials; Tetracycline (30 μg), Gentamicin (10 μg), Amikacin (30 μg), Ciprofloxacin (5 μg), Chloramphenicol (30 μg), Aztreonam (30 μg), Trimethoprim/sulfamethoxazole (1.25/23.75 μg), Amoxicillin-clavulanate (20/10 μg), Piperacillin/tazobactam (100/10 μg), Cefoxitin (30 μg), Ceftriaxone (30 μg), Imipenem (10 μg), Ertapenem (10 μg) and Meropenem (10 μg) (Oxoid, UK) and (BD, USA). After 16–18 hours of incubation at 35± 2°C, the diameter of the zone of inhibition around antibiotic discs was measured by caliper and interpreted as sensitive, intermediate, or resistant following CLSI (2018) guidelines [2].

### Phenotypic confirmatory test for carbapenemase

*K*. *pneumoniae* isolates that showed no sensitivity to at least one carbapenems were checked for carbapenemase production using the modified Carbapenem Inactivation Method (mCIM). According to CLSI (2018) guidelines the method has > 99% sensitivity and specificity for detection of carbapenemase among *Enterobacteriaceae* isolates [2]. Briefly, a suspension was made by taking 1μl loopful of bacteria from an overnight grown culture on a Blood agar plate and then added into 2 ml trypticase soya broth. Subsequently, Meropenem (10 μg) disc was immersed in the suspension and incubated for 4 hours ± 15 minutes at 35°C ± 2°C. After incubation, the disc was removed from the suspension using a 10 μl inoculation loop and placed on a Mueller-Hinton agar plate inoculated with a susceptible *E*. *coli* indicator strain (ATCC 29522). Then, the results were read after 18–24 hours of incubation at 35°C ± 2°C. When the bacterial isolate produced carbapenemase, the meropenem disc was inactivated allowing uninhibited growth of the susceptible indicator strain. Discs incubated in suspensions that do not contain carbapenemases yielded a clear inhibition zone. An inhibition zone diameter of 6–15 mm or colonies within a 16–18 mm zone was considered to be a positive result, and a zone of inhibition ≥19 mm was considered to be a negative result [2].

### DNA extraction and detection of *bla*_KPC_ and *bla*_NDM_ carbapenemase genes

The bacterial DNA was extracted by the boiling lysis method as previously described by El-Badawy et al [14]. Briefly, three to six fresh colonies of the bacteria were suspended in 100μl of DNase-free water in a sterile 1.5ml Eppendorf tube. The bacterial suspension was vortexed for 15 seconds and placed in a boiling Water-bath at 94°C for 10 minutes to lyse the bacterial cells. The lysed bacterial suspension was centrifuged at maximum speed (13,000 ×g) for 5 min. The supernatant, which contains total genomic DNA, was transferred to a new sterile tube using DNase-free tips. The quality and quantity of the extracted DNA were measured using Nanodrop (Thermo Scientific, US) and stored at -20^∘^C.

Multiplex PCR was performed to detect *bla*_KPC_ and *bla*_NDM_ carbapenemase genes using specific primers (Table 1). Briefly, the PCR was performed with approximately 300ng template DNA, 0.2μM of each primer, and 7.5 μl of 2 x QIAGEN Multiplex PCR Master Mix (QIAGEN, Germany) in a final volume of 15μl. Amplification was performed in a thermocycler (Biometra, Germany) with cycling parameters including initial denaturation at 95°C for 15 minutes followed by 35 cycles each of denaturation at 94°C for 30 s, annealing at 58°C for 90 s, extension at 72°C for 90 s, and a final extension at 72°C for 10 minutes. The PCR products were visualized by electrophoresis in 1.5% agarose gel after staining with ethidium bromide. A 100bp ladder molecular weight marker (Promega, US) was used to measure the molecular weight of amplified products. The amplicon was visualized and its size was determined under UV trans-illuminator (Bio-Rad, US).

**Table 1:** Primers used for detection of *bla*_KPC_ and *bla*_NDM_ carbapenemase genes in *K*. *pneumoniae* isolates.

Gene	Primer		Nucleotide sequence	Annealing Temp °C	Amplicon size (bp)	Reference
			5`————————-3`			
*bla*_KPC_	KPC	F	CGTCTAGTTCTGCTGTCTTG	57.8	798	[15]
		R	CTTGTCATCCTTGTTAGGCG	62.2		
*bla*_NDM_	NDM	F	GGTTTGGCGATCTGGTTTTC	65.5	621	
		R	CGGAATGGCTCATCACGATC	67.8		

### Data quality assurance

The reliability of the study findings was guaranteed by implementing quality control measures throughout the whole process of the laboratory work. Quality control strains of *Escherichia coli* ATCC® 25922 and *Pseudomonas aeruginosa* ATCC® 27853 were used for controlling the potency of the drugs. *K*. *pneumoniae* ATCC® BAA-1705™ and *K*. *pneumoniae* ATCC® BAA-1706 were used as positive and negative controls respectively during mCIM. Laboratory reference *bla*_KPC_ and *bla*_NDM_ genes were used as positive controls and *Escherichia coli* ATCC® 25922 as a negative control during PCR analysis. Each primer pair was checked in monoplex PCR before multiplexing.

### Data analysis

Data were checked, cleaned, and double entered into Epidata software version 3.1 (The EpiData Association, Denmark), and then it was exported to Statistical Package for Social Sciences (SPSS version 25.0, IBM Corp., USA) software for analysis. The chi-square test or Fisher’s exact test was used as appropriate. Bivariate logistic regression was carried out and variables with a *P*-value of less than 0.2 were entered into multivariate logistic regression analysis. A *P*-value < 0.05 at 95% confidence interval was considered as statistically significant.

### Ethical approval

This study was approved by the Ethics Review Committee of Department of Microbiology, Immunology, and Parasitology, School of Medicine, College of Health Sciences, Addis Ababa University (Reference number: DERC/17/18/02-N) and AHRI/ALERT ethical review committee (Protocol number: PO12/18). A permission letter was obtained from TASH. Moreover, before commencing the study, written informed consent/assent was obtained from each study participant. Confidentiality was maintained for all data collected.

## Results

### Prevalence of carbapenemase-producing *K*. *pneumoniae* isolates

**Fig 1:**
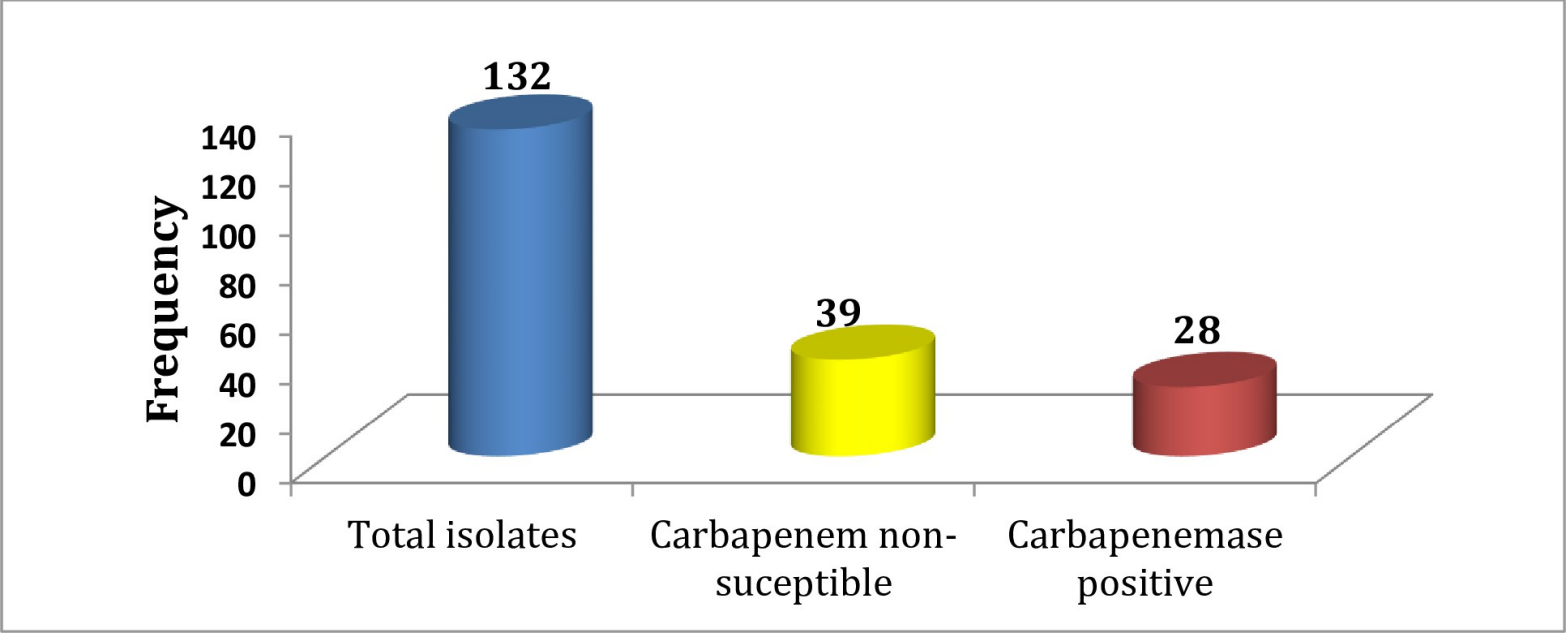
Frequency of carbapenemase-producing *K*. *pneumoniae* isolates.

**Fig 2:**
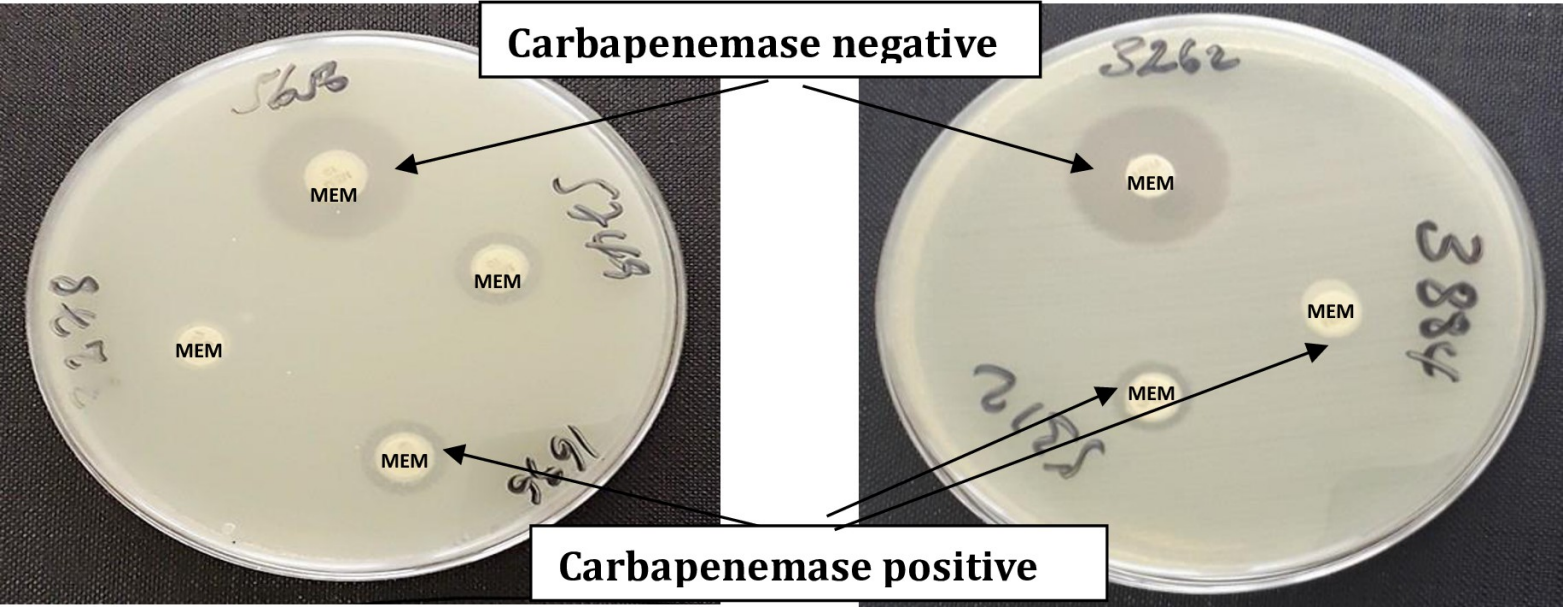
Carbapenemase positive and negative *K*. *pneumoniae* isolates with modified Carbapenem Inactivation Method (mCIM). MEM: Meropenem.

In this study, a total of 132 non-duplicate *K*. *pneumoniae* isolates were collected from patients who admitted or attended different departments of TASH. As shown in Fig 1 from the total isolates, 39 (29.6%) showed no sensitivity to one or more carbapenems. Out of these isolates, 28/39 (71.8%) were carbapenemase positive using the modified Carbapenem Inactivation Method (mCIM). The overall prevalence of carbapenemase production from the total isolates was 28/132 (21.2%). Fig 2 indicates the positive and negative results of mCIM.

### Distribution of carbapenemase-producing and non-producing isolates among age, sex, and ward type

Among the total *K*. *pneumoniae* isolates, 83/132 (62.9%) were recovered from males, of which 18 (21.7%) were carbapenemase positive. Regarding the age of study participants, the majority 74 (56.1%) were below 5 years. The majority of 120 (90.9%) *K*. *pneumoniae* isolates were recovered from hospitalized patients, of them, 27 (22.5%) were positive for carbapenemase (Table 2).

**Table 2:** Distribution of carbapenemase-producing and non-producing isolates among age, sex, and ward type.

Variables		Total	Carbapenemase	
			Positive n (%)	Negative n (%)
**Sex**	Male	83	18(21.7)	65(78.3)
	Female	49	10(20.4)	39(79.6)
**Age in years**	<5	74	7(9.5)	67(90.5)
	5 to <18	20	7(35.0)	13(65.0)
	18 to <45	25	11(44.0)	14(56.0)
	≥45	13	3(23.1)	10(76.9)
**Patient setting**	Inpatients	120	27(22.5)	93(77.5)
	Outpatient	12	1(8.3)	11(91.7)
**Ward type**	ICUs	46	8(17.4)	38(82.6)
	Pediatric ward	53	9(17.0)	44(83.0)
	Medical ward	9	6(66.7)	3(33.3)
	Surgical ward	8	4(50.0)	4(50.0)
	Others	4	0(0.0)	4(100.0)

n: number of *K*. *pneumoniae* isolates, ICUs: Intensive Care Units.

### Specimen-wise distribution of carbapenemase-producing *K*. *pneumoniae* isolates

**Fig 3:**
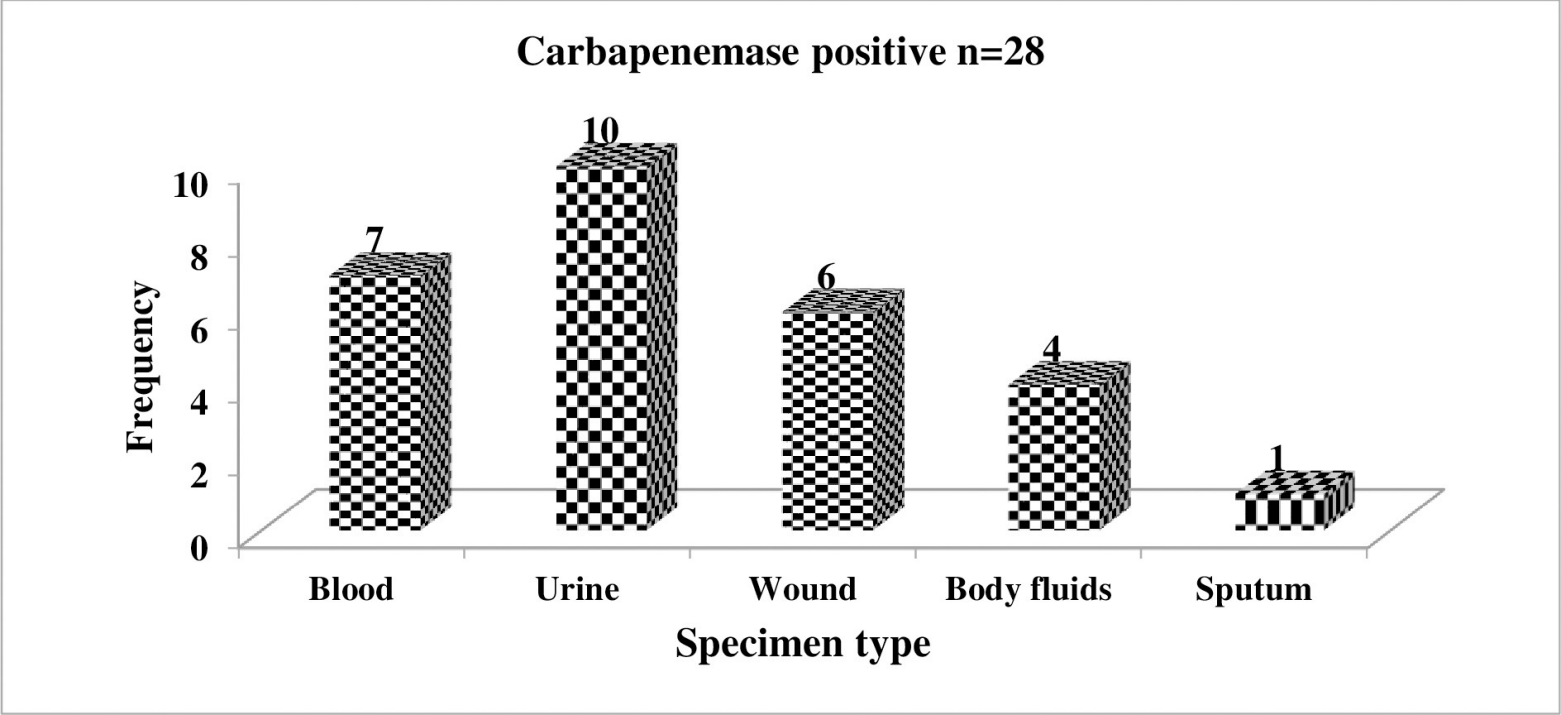
Specimen-wise distribution of carbapenemase-producing *K*. *pneumoniae* isolates.

As displayed in Fig 3, urine was the major source of carbapenemase-producing *K*. *pneumoniae* isolates with 10/28 (35.7%), while only 1/28 (3.6%) of carbapenemase-producing *K*. *pneumoniae* was isolated from sputum.

### Antimicrobial susceptibility patterns of carbapenemase-producing and non-producing *K*. *pneumoniae* isolates

Resistance of carbapenemase-producing isolates was high to β-lactams as well as other classes of antimicrobials except to amikacin. They were completely non-susceptible to aztreonam, piperacillin-tazobactam, amoxicillin-clavulanate, cefoxitin, ceftriaxone, meropenem, and ertapenem. The susceptibility of carbapenemase producers was 20/28 (7l.4%) to amikacin and from carbapenems least resistance was noted to imipenem 13/28 (46.4%). Carbapenemase positive isolates showed significantly higher resistance to most of the antimicrobials tested including ciprofloxacin (P<0.001), aztreonam (P = 0.016), piperacillin-tazobactam (P<0.001), chloramphenicol (p = 0.018), ceftriaxone (P<0.001), and carbapenems (P<0.001) compared to carbapenemase negative isolates with chi-square test as shown in Table 3. Almost all 130/132 (98.5) *K*. *pneumoniae* isolates were multidrug resistance (MDR). The details of susceptibility patterns of each *K*. *pneumoniae* isolate are presented in the S1 Table.

**Table 3:** Antimicrobial susceptibility patterns of carbapenemase-producing and non-producing *K*. *pneumoniae* isolates.

Antimicrobial agents	Carbapenemase positive (n = 28)			Carbapenemase negative (n = 104)			P-value
	S	I	R	S	I	R	
Tetracycline	5(17.9)	4(14.3)	19(67.9)	16(15.4)	11(10.6)	77(74.0)	0.791
Gentamicin	4(14.3)	0(0.0)	24(85.7)	25(24.0)	8(7.7)	71(68.3)	0.133
Amikacin	20(71.4)	4(14.3)	4(14.3)	103(99.0)	1(1.0)	0(0.0)	<0.001*
Ciprofloxacin	1(3.6)	2(7.1)	25(89.3)	57(54.8)	23(22.1)	24(23.1)	<0.001*
Aztreonam	0(0.0)	3(10.7)	25(89.3)	13(12.5)	27(26.0)	64(61.5)	0.016*
Piperacillin-tazobactam	0(0.0)	0(0.0)	28(100.0)	55(52.9)	27(26.0)	22(21.2)	<0.001*
Amoxicillin-clavulanate	0(0.0)	1(3.6)	27(96.4)	18(17.3)	31(29.8)	55(52.9)	<0.001*
SXT	3(10.7)	1(3.6)	24(85.7)	3(2.9)	1(1.0)	100(96.2)	0.121
Chloramphenicol	7(25.0)	10(35.7)	11(39.3)	52(50.0)	3(2.9)	49(47.1)	0.018*
Cefoxitin	0(0.0)	0(0.0)	28(100.0)	62(59.6)	12(11.5)	30(28.8)	<0.001*
Ceftriaxone	0(0.0)	0(0.0)	28(100.0)	4(3.8)	0(0.0)	100(96.2)	0.578
Meropenem	0(0.0)	3(10.7)	25(89.3)	96(92.3)	1(1.0)	7(6.7)	<0.001*
Imipenem	6(21.43)	9(32.1)	13(46.4)	101(97.1)	0(0.0)	3(2.9)	<0.001*
Ertapenem	0(0.0)	2(7.1)	26(92.9)	93(89.4)	3(2.9)	8(7.7)	<0.001*

S: Sensitive, I: Intermediate, R: Resistance, SXT: Trimethoprim-sulfamethoxazole

*P-value<0.05.

### Association of antimicrobial use with carbapenemase production

The possible association of history of antimicrobial consumption (within 3 months) with carbapenemase production was assessed by bivariate and multivariate logistic regression. Based on the local availability, the following antimicrobials were taken by the study participants in each class; 3^rd^ or 4^th^ generation cephalosporins (ceftriaxone, cefotaxime, ceftazidime, and cefepime), carbapenems (meropenem), quinolones (ciprofloxacin), aminoglycosides (gentamicin), and others such as trimethoprim-sulfamethoxazole, erythromycin, augmentin. As revealed in Table 4, from the total study participants, 83/132 (62.9%) have taken 3^rd^ or 4^th^ generation cephalosporins. Out of the total study participants, 20/132 (15.2%) have taken carbapenems of them, 11/20 (55%) were positive for carbapenemase. There was a statistically significant association between carbapenem use and carbapenemase production with multivariate logistic regression (P = 0.004).

**Table 4:** Association of antimicrobial use and carbapenemase production (N = 132).

Variable		CP	CN	COR (95%CI) P-value	AOR (95%CI) P-value*
Antimicrobial therapy	Yes	27	89	4.55 (0.57–36.05) 0.151	1.82 (0.19–17.91) 0.607
	No	1	15	1	1
Carbapenems	Yes	11	9	6.83 (2.46–18.96) <0.001	6.03 (2.13–17.09) **0.004**
	No	17	95	1	1
3^rd^ or 4^th^ GCs	Yes	21	62	2.03 (0.79–5.21) 0.140	1.67 (0.56–5.04) 0.478
	No	7	42	1	1
Quinolones	Yes	4	10	1.57 (0.45–5.43) 0.479	_
	No	24	94	1	
Aminoglycosides	Yes	12	31	1.77 (0.75–4.17) 0.194	1.70 (0.66–4.38) 0.538
	No	16	73	1	1

CP: Carbapenemase positive, CN: Carbapenemase negative, 3^rd^ or 4^th^ GCs: Third or fourth-generation cephalosporins, COR: Crude odds ratio, AOR: Adjusted odds ratio, CI: Confidence interval

* FDR adjusted P-value.

### Detection of *bla*_KPC_ and *bla*_NDM_ carbapenemase genes

**Fig 4:**
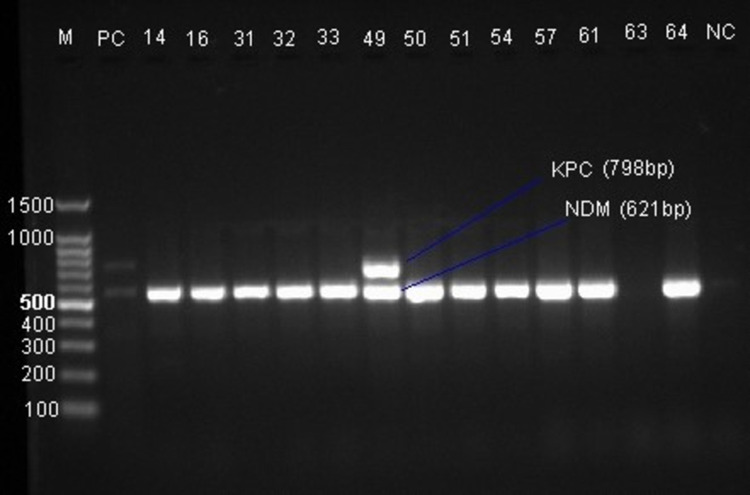
Agarose gel electrophoresis of PCR products for carbapenemase genes. Lane M: 100bp DNA ladder, PC: Positive control, Lanes 14–64: *K*. *pneumoniae* isolates, NC: Negative control.

Of the 28 carbapenemase-producing *K*. *pneumoniae* isolates with mCIM, 26 (92.9%) were positive for one or both carbapenemase genes in the multiplex PCR. The *bla*_NDM_ gene was detected in 26 isolates while the *bla*_KPC_ gene was detected in only one isolate concurrently with *bla*_NDM_. More than two-thirds (65.4%) of *bla*_NDM_ gene-positive isolates were from hospitalized patients in the ICU and pediatric wards (S2 Table). Fig 4 shows the gel image of the *bla*_KPC_ (798 bp), and *bla*_NDM_ (621 bp) genes.

## Discussion

There were few studies on carbapenemase-producing bacteria in Ethiopia and almost all of them noted *K*. *pneumoniae* as the most common carbapenemase producer compared to other bacterial isolates [16–18]. In the current study, 39 *K*. *pneumoniae* isolates were not sensitive to carbapenems, of them, 71.8% were carbapenemase positive phenotypically. Likewise, a study from Sudan showed that 78% of carbapenem-resistant *K*. *pneumoniae* isolates were carbapenemase positive [19]. In this study, the overall prevalence of carbapenemase-producing *K*. *pneumoniae* was 21.2%, which is comparable with another study conducted in Ethiopia from Bahir Dar (16.5%) [18]. It is lower than a study by Kazemian et al from Iran at which 43.3% of *K*. *pneumoniae* from hospitalized patients were carbapenemase producers [20]. Nevertheless, it is higher than a study from Tunisian and Libyan hospitals (11.4%) [21]. Empirical prescription of carbapenems particularly meropenem was very common in the hospital where we did the current study [22] and also in another study by Gebretekle et al a significant amount (38.6%) of meropenem was prescribed when it was not needed [23]. Additionally, in this study, previous carbapenem use has an association with carbapenemase production. This implies that the higher carbapenemase production in the current study could be due to selective pressure created by the indiscriminate use of carbapenems. Furthermore, it might be due to improper infection control practices as the emergence of carbapenemase-producing *K*. *pneumoniae* was previously noted in the hospital [17, 24]. A higher number of carbapenemase-producing *K*. *pneumoniae* was isolated from urine specimens 10/28 (35.7%), which is similar to a study conducted by Hashemizadeh et al [25].

In our study, almost all (98.5%) *K*. *pneumoniae* isolates were non-susceptible to at least three antimicrobials belonging to different categories and, hence, defined as MDR according to Magiorakos et al [26]. Concerning carbapenemase-producing isolates, relatively higher sensitivity (71.4%) was noted to amikacin, which is comparable with a study in Taiwan (78.8%) [27] suggesting the possible use of this drug against carbapenemase producers. Nonetheless, complete non-susceptibility of carbapenemase-producing *K*. *pneumoniae* isolates was observed to aztreonam, amoxicillin-clavulanate, piperacillin-tazobactam, cefoxitin, ceftriaxone, cefotaxime, ceftazidime, cefepime, meropenem, and ertapenem, which is in line with a report from Taiwan [27]. It is indicated that carbapenemase-producing Gram-negative bacteria, in particular, are resistant to all or virtually all beta-lactams, fluoroquinolones, and/or aminoglycosides concomitantly [1]. This is mainly due to the simultaneous presence of several resistance genes in these isolates. *K*. *pneumoniae* strains with a high prevalence of resistance against many antimicrobials including imipenem, amoxicillin/clavulanic acid, ceftazidime, piperacillin/tazobactam, tobramycin, ciprofloxacin, co-trimoxazole, and aztreonam, harboring genes encoding multi-drug efflux pump (AcrAB-TolC) and porins (OmpK35 and OmpK36) has been also reported [11, 28].

Karaiskos and Giamarellou (2014) described the worldwide emergence of carbapenemases mediated carbapenem resistance in *Klebsiella pneumoniae*, *Acinetobacter baumannii*, and *Pseudomonas aeruginosa* with a mortality exceeding 50%, is attributed mainly to the lack of effective antimicrobial regimens [29]. Generally, although they have limitations concerning accessibility, resistance, clinical efficacy, and adverse effects; colistin, tigecycline, fosfomycin, temocillin, and newer underdevelopment antimicrobials including carbapenemase inhibitors alone or in combination exhibit promising and/or effective antibacterial activity in vitro and some in vivo against infections caused by carbapenemase-producing bacteria [6, 29].

Two isolates that were positive by mCIM, harbored neither *bla*_KPC_ nor *bla*_NDM_ gene. This could be due to other carbapenemase genes, including *bla*_VIM_ and *bla*_IMP_ which belong to class B β-lactamases based on the Ambler classification system and *bla*_OXA-48_, a class D β-lactamase that are reported in *K*. *pneumoniae* increasingly [5, 8]. In this study, *bla*_NDM_ was detected in 26 out of 28 carbapenemase positive isolates which is the dominant carbapenemase-encoding gene compared to *bla*_KPC_ noticed only in one isolate. Similarly, in a study from Sudan, (70.7%) of *K*. *pneumoniae* isolates were positive for *bla*_NDM_ with no *bla*_KPC_ gene [30], and in a study from Egypt, *bla*_NDM-1_ was the most predominant carbapenemase gene in *K*. *pneumoniae (74*.*4%) compared to bla*_KPC_ (48.8%) [31]. The first reported *bla*_NDM-1_ in Kenya was in *K*. *pneumoniae and c*orresponds to the first report of NDM-1 producers in Africa [32]. There was also a previous report of *bla*_NDM-1_ positive *Acinetobacter baumannii* in Jimma, Ethiopia [11]. It has been described that *K*. *pneumoniae* is the most common species among *Enterobacteriaceae* that harbors *bla*_NDM_ and the rapid spread of the gene from its initial emergence in India to all continents is significantly associated with global travel [33]. Since Ethiopians make travel to India and other countries for medical purposes and other reasons there is a possibility to acquire carbapenemase genes. However, it is difficult to conclude without taking a detailed history of patients. Although there are high-risk KPC-carrying *K*. *pneumoniae clones such as* ST258 and ST11 as well as most common NDM positive *K*. *pneumoniae* lineages such as ST11 and ST14 [33], this study has limitations in that sequencing wasn’t done due to resource constraint.

## Conclusion and recommendations

In this study, the prevalence of carbapenemase-producing *K*. *pneumoniae* isolates was a matter of great concern. Carbapenemase-producing isolates were highly resistant to many of the antimicrobials used in this study. Only amikacin was relatively active against carbapenemase-producing isolates. Moreover, previous use of carbapenems was associated with carbapenemase production suggesting the need to implement effective antimicrobial stewardship practices in the hospital. To our knowledge, the study described the emergence of *bla*_NDM_ and *bla*_KPC_ gene carrying *K*. *pneumoniae* in Ethiopia for the first time. Further large-scale molecular-based studies, including other carbapenemase genes and sequencing of *K*. *pneumoniae* are warranted, to have a clear awareness about the presence of antimicrobial resistance high-risk clones in Ethiopia.

## Supporting information

S1 Table Antimicrobial susceptibility pattern and MDR status of 132 *K. pneumoniae* isolates at Tikur Anbessa Specialized Hospital. S: Sensitive, I: Intermediate, R: Resistant, TET: Tetracycline, GM: Gentamicin, AN: Amikacin, CIP: Ciprofloxacin, ATM: Aztreonam, PTZ: Piperacillin-tazobactam, AMC: Amoxicillin-clavulanate, SXT: Trimethoprim-sulfamethoxazole, CHL: Chloramphenicol, CXT: Cefoxitin, CRO: Ceftriaxone, MEM: Meropenem, IMP: Imipenem, ETM: Ertapenem.
(PDF)

S2 Table Patient characteristics, mCIM test result, ESBL result and carbapenemase genes of 39 carbapenem non-susceptible *K. pneumoniae* isolates at Tikur Anbessa Specialized Hospital. mCIM: modified Carbapenem Inactivation Method, ESBL: Extended-spectrum β-lactamase, Interp: Interpretation, ZI in mm: Zone of inhibition in millimeter, M: Male, F: Female, CSF: Cerebrospinal fluid, ICU: Intensive Care Unit.
(PDF)

S1 Fig Agarose gel electrophoresis of PCR products for carbapenemase genes. Lane M: 100bp DNA ladder; PC: Positive control, Lanes 67–114: *K*. *pneumoniae* isolates, NC: Negative control.
(TIF)

S2 Fig Agarose gel electrophoresis of PCR products for carbapenemase genes. Lane M: 100bp DNA ladder, PC1, PC2 & PC3: Positive control, Lanes 67–120: *K*. *pneumoniae* isolates, NC: Negative control.
(TIF)

## References

[1] Meletis G. Carbapenem resistance: overview of the problem and future perspectives. Ther Adv Infect Dis. 2016;3(1):15–21. doi: 10.1177/2049936115621709 26862399 PMC4735501

[2] CLSI. Performance standards for antimicrobial susceptibility testing. 28th ed. CLSI supplement M100-S28.Wayne, PA. Clinical and Laboratory Standards Institute 2018.

[3] Ranjbar R, Memariani H, Sorouri R, Memariani M. Distribution of virulence genes and genotyping of CTX-M-15-producing *Klebsiella pneumoniae* isolated from patients with community-acquired urinary tract infection (CA-UTI). Microb Pathog. 2016;100:244–249. doi: 10.1016/j.micpath.2016.10.002 27725280

[4] Harada S, Ishii Y, Yamaguchi K. Extended-spectrum β-lactamases: implications for the clinical laboratory and therapy. Korean J Lab Med. 2008;28(6):401–12. doi: 10.3343/kjlm.2008.28.6.401 19127103

[5] Logan LK, Weinstein RA. The Epidemiology of Carbapenem-Resistant *Enterobacteriaceae*: The Impact and Evolution of a Global Menace. J Infect Dis. 2017;215(suppl_1):S28–S36. doi: 10.1093/infdis/jiw282 28375512 PMC5853342

[6] Pitout JD, Nordmann P, Poirel L. Carbapenemase-producing *Klebsiella pneumoniae*, a key pathogen set for global nosocomial dominance. Antimicrob Agents Chemother. 2015;59(10):5873–84. doi: 10.1128/AAC.01019-15 26169401 PMC4576115

[7] Aslam B, Rasool M, Muzammil S, Siddique AB, Nawaz Z, Shafique M, et al. Carbapenem resistance: Mechanisms and drivers of global menace. Pathog Bact. 2020.

[8] Walsh TR. Emerging carbapenemases: a global perspective. Int J Antimicrob Agents. 2010;36:S8–S14. doi: 10.1016/S0924-8579(10)70004-2 21129630

[9] Nordmann P, Naas T, Poirel L. Global spread of carbapenemase-producing *Enterobacteriaceae*. Emerg Infect Dis. 2011;17(10):1791. doi: 10.3201/eid1710.110655 22000347 PMC3310682

[10] Ranjbar R, Izadi M, Hafshejani TT, Khamesipour F. Molecular detection and antimicrobial resistance of *Klebsiella pneumoniae* from house flies (Musca domestica) in kitchens, farms, hospitals and slaughterhouses. J Infect Public Health. 2016;9(4):499–505. doi: 10.1016/j.jiph.2015.12.012 26876433

[11] Pritsch M, Zeynudin A, Messerer M, Baumer S, Liegl G, Schubert S, et al. First report on bla NDM-1-producing *Acinetobacter baumannii* in three clinical isolates from Ethiopia. BMC Infect Dis. 2017;17(1):1–7. doi: 10.1186/s12879-016-2122-x 28249575 PMC5333390

[12] Cheesbrough M. Cheesbrough M. District laboratory practice in tropical countries: Part 2. 2^nd^ ed. New York: Cambridge University Press; 2006.

[13] Ranjbar R, Kelishadrokhi AF, Chehelgerdi M. Molecular characterization, serotypes and phenotypic and genotypic evaluation of antibiotic resistance of the *Klebsiella pneumoniae* strains isolated from different types of hospital-acquired infections. Infect Drug Resis. 2019;12:603. doi: 10.2147/IDR.S199639 31114256 PMC6489651

[14] El-Badawy MF, Tawakol WM, El-Far SW, Maghrabi IA, Al-Ghamdi SA, Mansy MS, et al. Molecular identification of aminoglycoside-modifying enzymes and plasmid-mediated quinolone resistance genes among *Klebsiella pneumoniae* clinical isolates recovered from Egyptian patients. Int J Microbiol. 2017;2017:8050432. doi: 10.1155/2017/8050432 28638412 PMC5468591

[15] Poirel L, Walshb RT, Cuvilliera V, Nordmanna P. Multiplex PCR for detection of acquired carbapenemase genes. Diagn Microbiol Infect Dis. 2011;70:119–23. doi: 10.1016/j.diagmicrobio.2010.12.002 21398074

[16] Eshetie S, Unakal C, Gelaw A, Ayelign B, Endris M, Moges F. Multidrug resistant and carbapenemase producing *Enterobacteriaceae* among patients with urinary tract infection at referral Hospital, Northwest Ethiopia. Antimicrob Resist Infect Control. 2015;4(1):1–8. doi: 10.1186/s13756-015-0054-7 25908966 PMC4407313

[17] Legese MH, Weldearegay GM, Asrat D. Extended-spectrum beta-lactamase-and carbapenemase-producing *Enterobacteriaceae* among Ethiopian children. Infect Drug Resis. 2017;10:27–34. doi: 10.2147/IDR.S127177 28182124 PMC5279835

[18] Moges F, Eshetie S, Abebe W, Mekonnen F, Dagnew M, Endale A, et al. High prevalence of extended-spectrum beta-lactamase-producing Gram-negative pathogens from patients attending Felege Hiwot Comprehensive Specialized Hospital, Bahir Dar, Amhara region. PLoS One. 2019;14(4):e0215177. doi: 10.1371/journal.pone.0215177 30986262 PMC6464180

[19] Dirar M, Bilal N, Ibrahim ME, Hamid M. Resistance Patterns and Phenotypic Detection of β-lactamase Enzymes among *Enterobacteriaceae* Isolates from Referral Hospitals in Khartoum State, Sudan. Cureus. 2020;12(3). doi: 10.7759/cureus.7260 32195070 PMC7075475

[20] Kazemian H, Heidari H, Ghanavati R, Ghafourian S, Yazdani F, Sadeghifard N, et al. Phenotypic and Genotypic Characterization of ESBL-, AmpC-, and Carbapenemase-Producing *Klebsiella pneumoniae* and *Escherichia coli* Isolates. Med Princ Pract. 2019;28(6):547–51. doi: 10.1159/000500311 30995662 PMC6944897

[21] Mathlouthi N, Al-Bayssari C, El Salabi A, Bakour S, Gwierif SB, Zorgani AA, et al. Carbapenemases and extended-spectrum β-lactamases producing *Enterobacteriaceae* isolated from Tunisian and Libyan hospitals. J Infect Dev Ctries. 2016;10(07):718–27. doi: 10.3855/jidc.7426 27482803

[22] Fenta T, Engidawork E, Amogne W, Berha AB. Evaluation of current practice of antimicrobial use and clinical outcome of patients with pneumonia at a tertiary care hospital in Ethiopia: A prospective observational study. PLoS One. 2020;15(1):e0227736. doi: 10.1371/journal.pone.0227736 31999752 PMC6992215

[23] Gebretekle GB, Haile Mariam D, Abebe Taye W, Mulu Fentie A, Amogne Degu W, Alemayehu T, et al. Half of Prescribed Antibiotics Are Not Needed: A Pharmacist-Led Antimicrobial Stewardship Intervention and Clinical Outcomes in a Referral Hospital in Ethiopia. Front public health. 2020;8:109. doi: 10.3389/fpubh.2020.00109 32328474 PMC7160317

[24] Desta K, Woldeamanuel Y, Azazh A, Mohammod H, Desalegn D, Shimelis D, et al. High gastrointestinal colonization rate with extended-Spectrum β-lactamase-producing *Enterobacteriaceae* in hospitalized patients: emergence of Carbapenemase-Producing *K*. *pneumoniae* in Ethiopia. PLoS One. 2016;11(8):e0161685. doi: 10.1371/journal.pone.0161685 27574974 PMC5004900

[25] Hashemizadeh Z, Hosseinzadeh Z, Azimzadeh N, Motamedifar M. Dissemination Pattern of Multidrug Resistant Carbapenemase Producing *Klebsiella pneumoniae* Isolates Using Pulsed-Field Gel Electrophoresis in Southwestern Iran. Infect Drug Resist. 2020;13:921. doi: 10.2147/IDR.S227955 32280248 PMC7125322

[26] Magiorakos AP, Srinivasan A, Carey R, Carmeli Y, Falagas M, Giske C, et al. Multidrug‐resistant, extensively drug‐resistant and pandrug‐resistant bacteria: An international expert proposal for interim standard definitions for acquired resistance. Clin Microbiol Infect. 2012; 18: 268–281. doi: 10.1111/j.1469-0691.2011.03570.x 21793988

[27] Chiu SK, Ma L, Chan MC, Lin YT, Fung CP, Wu TL, et al. Carbapenem nonsusceptible *Klebsiella pneumoniae* in Taiwan: dissemination and increasing resistance of carbapenemase producers during 2012–2015. Sci Rep. 2018;8(1):8468. doi: 10.1038/s41598-018-26691-z 29855588 PMC5981607

[28] Shakib P, Ghafourian S, Zolfaghary MR, Hushmandfar R, Ranjbar R, Sadeghifard N. Prevalence of OmpK35 and OmpK36 porin expression in beta-lactamase and non-betalactamase-producing *Klebsiella pneumoniae*. Biologics. 2012;6:1. doi: 10.2147/BTT.S27582 22291461 PMC3266860

[29] Karaiskos I, Giamarellou H. Multidrug-resistant and extensively drug-resistant Gram-negative pathogens: current and emerging therapeutic approaches. Expert Opin Pharmacother. 2014;15(10):1351–70. doi: 10.1517/14656566.2014.914172 24766095 PMC4819585

[30] Elbadawi HS, Elhag KM, Mahgoub E, Altayb HN, Ntoumi F, Elton L, et al. Detection and characterization of carbapenem resistant Gram‐negative bacilli isolates recovered from hospitalized patients at Soba University Hospital, Sudan. BMC Microbiol. 2021;21(1):1–9. doi: 10.1186/s12866-020-02060-7 33947325 PMC8094518

[31] Hammad HA, Hadiya S, El-Feky MA, Aly SA. Co-occurrence of Plasmid-mediated Quinolone Resistance and Carbapenemases in *Klebsiella pneumoniae* Isolates in Assiut, Egypt. Egypt J Med Microbiol. 2017;38(5792):1–7.

[32] Poirel L, Revathi G, Bernabeu S, Nordmann P. Detection of NDM-1-producing *Klebsiella pneumoniae* in Kenya. Antimicrob Agents Chemother. 2011;55(2):934–6. doi: 10.1128/AAC.01247-10 21115785 PMC3028766

[33] Wu W, Feng Y, Tang G, Qiao F, McNally A, Zong Z. NDM metallo-β-lactamases and their bacterial producers in health care settings. Clin Microbiol Rev. 2019;32(2). doi: 10.1128/CMR.00115-18 30700432 PMC6431124

