# Supplementary material for: Detection of blaKPC and blaNDM carbapenemase genes among Klebsiella pneumoniae isolates in Addis Ababa, Ethiopia: Dominance of blaNDM
Source: PLoS One. 2022 Apr 27;17(4):e0267657. doi: 10.1371/journal.pone.0267657 (PMC9045624; doi:10.1371/journal.pone.0267657)
Supplement: S1 Table — S: Sensitive, I: Intermediate, R: Resistant, TET: Tetracycline, GM: Gentamicin, AN: Amikacin, CIP: Ciprofloxacin, ATM: Aztreonam, PTZ: Piperacillin-tazobactam, AMC: Amoxicillin-clavulanate, SXT: Trimethoprim-sulfamethoxazole, CHL: Chloramphenicol, CXT: Cefoxitin, CRO: Ceftriaxone, MEM: Meropenem, IMP: Imipenem, ETM: Ertapenem. (PDF) [file pone.0267657.s001.pdf]

S1 Table. Antimicrobial susceptibility pattern and MDR status of 132 *K. pneumoniae* isolates at Tikur Anbessa Specialized Hospital

Code	TET	GM	AN	CIP	ATM	PTZ	AMC	SXT	CHL	CXT	CRO	MEM	IMP	ETM	MDR Status
**1**	11	8	23	17	19	13	6	6	23	18	6	30	28	24	MDR
	R	R	S	R	I	R	R	R	S	S	R	S	S	S	
**2**	7	8	22	26	19	12	10	6	25	20	6	28	26	26	MDR
	R	R	S	S	I	R	R	R	S	S	R	S	S	S	
**3**	12	6	22	16	17	13	6	6	23	17	6	28	27	23	MDR
	I	R	S	I	R	R	R	R	S	I	R	S	S	S	
**4**	6	12	19	26	19	24	18	6	21	18	6	23	24	27	MDR
	R	R	S	S	I	S	S	R	S	S	R	S	S	S	
**5**	15	10	27	22	15	23	15	6	22	16	10	31	25	25	MDR
	S	R	S	S	R	S	I	R	S	I	R	S	S	S	
**6**	12	8	22	21	18	18	12	6	22	20	6	26	26	23	MDR
	I	R	S	S	I	I	R	R	S	S	R	S	S	S	
**7**	6	8	21	20	19	23	6	6	6	19	6	29	28	28	MDR
	R	R	S	S	I	S	R	R	S	S	R	S	S	S	
**8**	17	10	23	6	8	21	13	6	23	16	6	31	26	25	MDR
	S	R	S	R	R	S	R	R	S	I	R	S	S	S	
**9**	6	11	28	28	10	23	16	6	15	16	6	29	27	23	MDR
	R	R	S	S	R	S	I	R	I	I	R	S	S	S	
**10**	6	11	23	17	13	22	12	6	6	19	6	30	25	24	MDR
	R	R	S	I	R	S	R	R	R	S	R	S	S	S	
**11**	16	15	25	9	13	20	15	6	6	17	6	25	26	26	MDR
	S	S	S	R	R	S	I	R	R	I	R	S	S	S	
**12**	6	13	23	21	19	22	6	6	6	6	12	24	24	24	MDR
	R	I	S	S	I	S	R	R	R	R	R	S	S	S	
**13**	6	12	21	6	20	21	11	6	6	19	6	27	28	23	MDR
	R	R	S	R	I	S	R	R	R	S	R	S	S	S	

Zone of inhibition in millimeter (mm)

Interpretation: S: Sensitive; I:Intermidate; R: Resistant

TET: Tetracycline; GN: Gentamicin: Amikacin; CIP: Ciprofloxacin; ATM: Aztreonam; PTZ: Piperacillin/tazobactam; AMC: Amoxicillin-clavulanate; SXT:Trimethoprim/sulfamethoxazole; CHL: Chloramphenicol; CXT: Cefoxitin; CRO: Ceftriaxone; MEM: Meropenem; IMP: Imipenem; ETM: Ertapenem

**14**	16	7	17	6	6	14	10	6	6	6	6	8	23	6	MDR
	S	R	S	R	R	R	R	R	R	R	R	R	S	R	
**15**	6	11	22	18	17	19	18	6	6	21	9	25	28	28	MDR
	R	R	S	I	R	I	S	R	R	S	R	S	S	S	
**16**	6	12	25	6	6	13	6	6	16	6	6	13	20	6	MDR
	R	R	S	R	R	R	R	R	I	R	R	R	I	R	
**17**	6	12	25	16	6	20	12	6	25	21	6	29	28	23	MDR
	R	R	S	I	R	I	R	R	S	S	R	S	S	S	
**18**	8	13	23	19	20	23	7	6	6	13	11	29	28	24	MDR
	R	I	S	I	I	S	R	R	R	R	R	S	S	S	
**19**	18	6	24	27	7	19	6	6	6	18	6	31	26	25	MDR
	S	R	S	S	R	I	R	R	R	S	R	S	S	S	
**20**	11	15	19	14	6	17	9	6	6	6	6	24	24	19	MDR
	R	S	S	R	R	R	R	R	R	R	R	S	S	I	
**21**	11	24	25	28	22	14	9	20	27	6	16	25	27	25	MDR
	R	S	S	S	S	R	R	S	S	R	R	S	S	S	
**22**	6	12	24	26	14	20	11	6	6	20	6	24	26	25	MDR
	R	R	S	S	R	I	R	R	R	S	R	S	S	S	
**23**	16	12	27	29	14	20	13	6	14	17	6	29	23	23	MDR
	S	R	S	S	R	I	R	R	I	I	R	S	S	S	
**24**	13	10	20	19	16	19	6	6	23	6	8	25	24	22	MDR
	I	R	S	I	R	I	R	R	S	R	R	S	S	S	
**25**	6	11	23	24	16	22	15	14	6	19	8	26	26	26	MDR
	R	R	S	S	R	S	I	I	R	S	R	S	S	S	
**26**	6	15	27	18	19	21	14	6	6	21	11	33	29	26	MDR
	R	S	S	I	I	S	I	R	R	S	R	S	S	S	
**27**	6	10	20	15	8	18	6	6	6	6	6	24	26	20	MDR
	R	R	S	R	R	I	R	R	R	R	R	S	S	I	
**28**	6	14	27	18	20	23	16	6	6	24	11	32	29	25	MDR
	R	I	S	I	S	S	I	R	R	S	R	S	S	S	
**29**	10	9	23	25	12	18	10	6	21	16	6	27	25	22	MDR

Zone of inhibition in millimeter (mm)

Interpretation: S: Sensitive; I:Intermidate; R: Resistant

TET: Tetracycline; GN: Gentamicin: Amikacin; CIP: Ciprofloxacin; ATM: Aztreonam; PTZ: Piperacillin/tazobactam; AMC: Amoxicillin-clavulanate; SXT:Trimethoprim/sulfamethoxazole; CHL: Chloramphenicol; CXT: Cefoxitin; CRO: Ceftriaxone; MEM: Meropenem; IMP: Imipenem; ETM: Ertapenem

	R	R	S	S	R	I	R	R	S	I	R	S	S	S	
**30**	6	21	21	6	15	21	12	6	26	20	6	28	24	24	MDR
	R	S	S	R	R	S	R	R	S	S	R	S	S	S	
**31**	15	10	19	6	6	6	6	6	17	6	6	6	19	6	MDR
	S	R	S	R	R	R	R	R	I	R	R	R	R	R	
**32**	10	6	18	21	12	13	6	6	24	10	9	21	22	18	MDR
	R	R	S	S	R	R	R	R	S	R	R	I	I	R	
**33**	15	10	16	6	6	6	6	6	21	6	6	6	17	6	MDR
	S	R	I	R	R	R	R	R	S	R	R	R	R	R	
**34**	13	6	20	6	14	16	8	6	6	6	6	23	25	23	MDR
	I	R	S	R	R	R	R	R	R	R	R	S	S	S	
**35**	8	10	26	25	18	25	6	6	18	6	8	28	27	25	MDR
	R	R	S	S	I	S	R	R	S	R	R	S	S	S	
**36**	16	14	26	23	15	23	6	26	23	6	6	28	28	26	MDR
	S	I	S	S	R	S	R	S	S	R	R	S	S	S	
**37**	6	17	24	15	10	24	18	6	6	19	8	29	31	26	MDR
	R	S	S	R	R	S	S	R	R	S	R	S	S	S	
**38**	6	12	26	29	6	15	12	6	6	8	6	23	26	23	MDR
	R	R	S	S	R	R	R	R	R	R	R	S	S	S	
**39**	15	13	22	6	6	12	20	6	6	6	6	12	23	11	MDR
	S	I	S	R	R	R	S	R	R	R	R	R	S	R	
**40**	6	22	22	22	16	22	11	6	24	18	8	23	25	24	MDR
	R	S	S	S	R	S	R	R	S	S	R	S	S	S	
**41**	6	8	22	17	15	19	11	6	6	20	6	23	26	27	MDR
	R	R	S	I	R	I	R	R	R	S	R	S	S	S	
**42**	6	8	16	20	10	17	6	6	16	10	6	29	30	26	MDR
	R	R	I	I	R	R	R	R	I	R	R	S	S	S	
**43**	6	12	22	17	6	17	6	6	6	10	6	28	29	27	MDR
	R	R	S	I	R	R	R	R	R	R	R	S	S	S	
**44**	6	22	22	15	8	19	16	6	6	12	6	29	27	24	MDR
	R	S	S	R	R	I	I	R	R	R	R	S	S	S	

Zone of inhibition in millimeter (mm)

Interpretation: S: Sensitive; I:Intermidate; R: Resistant

TET: Tetracycline; GN: Gentamicin: Amikacin; CIP: Ciprofloxacin; ATM: Aztreonam; PTZ: Piperacillin/tazobactam; AMC: Amoxicillin-clavulanate; SXT:Trimethoprim/sulfamethoxazole; CHL: Chloramphenicol; CXT: Cefoxitin; CRO: Ceftriaxone; MEM: Meropenem; IMP: Imipenem; ETM: Ertapenem

**45**	17	21	22	17	19	21	16	6	24	22	6	26	27	24	MDR
	S	S	S	I	I	S	I	R	S	S	R	S	S	S	
**46**	11	6	19	27	6	20	17	6	12	18	6	27	27	23	MDR
	R	R	S	S	R	I	I	R	R	S	R	S	S	S	
**47**	18	6	17	20	10	22	17	6	24	20	6	26	28	25	MDR
	S	R	S	I	R	S	I	R	S	S	R	S	S	S	
**48**	10	12	20	6	6	19	15	6	6	21	6	26	26	23	MDR
	R	R	S	R	R	S	I	R	R	S	R	S	S	S	
**49**	12	6	11	6	6	6	17	6	6	6	6	21	20	12	MDR
	I	R	R	R	R	R	I	R	R	R	R	I	I	R	
**50**	8	6	6	6	6	6	6	19	6	6	6	8	21	6	MDR
	R	R	R	R	R	R	R	S	R	R	R	R	I	R	
**51**	13	6	14	6	6	6	6	6	6	6	6	6	6	6	MDR
	I	R	R	R	R	R	R	R	R	R	R	R	R	R	
**52**	6	6	18	24	22	22	18	6	6	21	16	26	28	25	MDR
	R	R	S	S	S	S	S	R	R	S	R	S	S	S	
**53**	13	7	17	18	18	18	15	6	6	22	6	27	26	24	MDR
	I	R	S	I	I	I	I	R	R	S	R	S	S	S	
**54**	12	6	18	6	6	8	6	6	17	6	6	6	6	6	MDR
	I	R	S	R	R	R	R	R	I	R	R	R	R	R	
**55**	15	6	18	15	6	18	13	6	10	19	6	26	27	23	MDR
	S	R	S	R	R	I	R	R	R	S	R	S	S	S	
**56**	24	18	20	6	6	6	6	6	6	6	6	6	6	6	MDR
	S	S	S	R	R	R	R	R	R	R	R	R	R	R	
**57**	15	7	21	6	6	6	6	6	19	6	6	6	6	6	MDR
	S	R	S	R	R	R	R	R	S	R	R	R	R	R	
**58**	9	16	18	24	14	21	16	6	21	20	6	27	27	24	MDR
	R	S	S	S	R	S	I	R	S	S	R	S	S	S	
**59**	11	6	17	16	12	19	14	6	25	23	10	27	26	24	MDR
	R	R	S	I	R	I	I	R	S	S	R	S	S	S	
**60**	6	9	23	25	22	23	15	6	10	23	6	25	26	30	MDR

Zone of inhibition in millimeter (mm)

Interpretation: S: Sensitive; I:Intermidate; R: Resistant

TET: Tetracycline; GN: Gentamicin: Amikacin; CIP: Ciprofloxacin; ATM: Aztreonam; PTZ: Piperacillin/tazobactam; AMC: Amoxicillin-clavulanate; SXT:Trimethoprim/sulfamethoxazole; CHL: Chloramphenicol; CXT: Cefoxitin; CRO: Ceftriaxone; MEM: Meropenem; IMP: Imipenem; ETM: Ertapenem

	R	R	S	S	S	S	I	R	R	S	R	S	S	S	
**61**	15	18	18	6	18	6	6	6	19	6	6	6	18	6	MDR
	S	S	S	R	I	R	R	R	S	R	R	R	R	R	
**62**	6	22	25	26	23	24	22	6	6	23	6	27	28	30	MDR
	R	S	S	S	S	S	S	R	R	S	R	S	S	S	
**63**	6	9	23	6	6	6	6	6	16	6	6	6	21	6	MDR
	R	R	S	R	R	R	R	R	I	R	R	R	I	R	
**64**	6	7	18	6	6	6	6	6	15	6	6	6	19	6	MDR
	R	R	S	R	R	R	R	R	I	R	R	R	R	R	
**65**	12	8	22	23	22	22	18	6	25	22	6	23	25	25	MDR
	I	R	S	S	S	S	S	R	S	S	R	S	S	S	
**66**	6	10	20	15	18	19	12	6	8	20	6	23	25	25	MDR
	R	R	S	R	I	I	R	R	R	S	R	S	S	S	
**67**	6	6	6	6	8	12	6	16	10	6	6	12	20	16	MDR
	R	R	R	R	R	R	R	S	R	R	R	R	I	R	
**68**	6	22	22	6	18	21	13	6	24	21	6	26	28	31	MDR
	R	S	S	R	I	S	R	R	S	S	R	S	S	S	
**69**	11	20	20	26	28	22	20	22	24	20	23	23	26	25	Not MDR
	R	S	S	S	S	S	S	S	S	S	S	S	S	S	
**70**	6	11	22	17	9	15	8	6	6	7	6	17	26	20	MDR
	R	R	S	I	R	R	R	R	R	R	R	R	S	I	
**71**	6	21	23	22	19	24	18	6	26	20	6	26	27	28	MDR
	R	S	S	S	I	S	S	R	S	S	R	S	S	S	
**72**	6	19	22	22	20	24	20	6	25	20	6	24	28	26	MDR
	R	S	S	S	I	S	S	R	S	S	R	S	S	S	
**73**	6	8	24	21	6	20	17	6	6	6	6	24	26	23	MDR
	R	R	S	S	R	I	I	R	R	R	R	S	S	S	
**74**	14	6	16	6	6	6	6	6	6	6	6	10	12	6	MDR
	I	R	I	R	R	R	R	R	R	R	R	R	R	R	
**75**	6	8	22	24	8	17	11	6	6	15	6	24	26	24	MDR
	R	R	S	S	R	R	R	R	R	I	R	S	S	S	

Zone of inhibition in millimeter (mm)

Interpretation: S: Sensitive; I:Intermidate; R: Resistant

TET: Tetracycline; GN: Gentamicin: Amikacin; CIP: Ciprofloxacin; ATM: Aztreonam; PTZ: Piperacillin/tazobactam; AMC: Amoxicillin-clavulanate; SXT:Trimethoprim/sulfamethoxazole; CHL: Chloramphenicol; CXT: Cefoxitin; CRO: Ceftriaxone; MEM: Meropenem; IMP: Imipenem; ETM: Ertapenem

**76**	6	10	22	10	6	19	10	6	22	16	6	24	26	24	MDR
	R	R	S	R	R	I	R	R	S	I	R	S	S	S	
**77**	6	8	24	24	12	20	11	6	24	21	6	25	28	29	MDR
	R	R	S	S	R	I	R	R	S	S	R	S	S	S	
**78**	10	7	21	23	10	15	14	6	6	15	6	23	26	24	MDR
	R	R	S	S	R	R	I	R	R	I	R	S	S	S	
**79**	16	11	23	22	21	24	6	6	6	6	6	25	25	27	MDR
	S	R	S	S	S	S	R	R	R	R	R	S	S	S	
**80**	6	6	23	26	13	23	14	6	28	21	6	25	26	28	MDR
	R	R	S	S	R	S	I	R	S	S	R	S	S	S	
**81**	6	20	17	6	18	6	6	6	17	6	6	6	18	6	MDR
	R	S	S	R	I	R	R	R	I	R	R	R	R	R	
**82**	8	22	23	30	21	23	8	6	26	6	6	27	28	28	MDR
	R	S	S	S	I	S	R	R	S	R	R	S	S	S	
**83**	7	6	16	6	6	17	10	14	11	12	6	20	25	21	MDR
	R	R	I	R	R	R	R	I	R	R	R	I	S	I	
**84**	12	7	20	22	11	20	9	6	23	17	6	23	26	26	MDR
	I	R	S	S	R	I	R	R	S	I	R	S	S	S	
**85**	12	8	22	23	18	24	18	6	25	21	6	25	27	27	MDR
	I	R	S	S	I	S	S	R	S	S	R	S	S	S	
**86**	6	14	24	26	18	22	6	6	27	6	6	23	24	24	MDR
	R	I	S	S	I	S	R	R	S	R	R	S	S	S	
**87**	6	19	17	18	13	17	6	6	22	6	6	18	21	17	MDR
	R	S	S	I	R	R	R	R	S	R	R	R	I	R	
**88**	6	10	26	28	13	21	16	6	25	22	6	27	26	23	MDR
	R	R	S	S	R	S	I	R	S	S	R	S	S	S	
**89**	18	20	21	22	20	24	20	6	22	17	26	24	26	24	MDR
	S	S	S	S	I	S	S	R	S	I	S	S	S	S	
**90**	6	7	18	6	6	6	6	6	16	6	6	6	14	6	MDR
	R	R	S	R	R	R	R	R	I	R	R	R	R	R	
**91**	6	19	22	19	19	21	15	6	26	19	11	24	28	27	MDR

Zone of inhibition in millimeter (mm)

Interpretation: S: Sensitive; I:Intermidate; R: Resistant

TET: Tetracycline; GN: Gentamicin: Amikacin; CIP: Ciprofloxacin; ATM: Aztreonam; PTZ: Piperacillin/tazobactam; AMC: Amoxicillin-clavulanate; SXT:Trimethoprim/sulfamethoxazole; CHL: Chloramphenicol; CXT: Cefoxitin; CRO: Ceftriaxone; MEM: Meropenem; IMP: Imipenem; ETM: Ertapenem

	R	S	S	I	I	S	I	R	S	S	R	S	S	S	
**92**	6	20	21	22	17	23	19	6	24	18	8	24	24	25	MDR
	R	S	S	S	R	S	S	R	S	S	R	S	S	S	
**93**	8	6	17	6	6	17	8	19	10	12	6	19	24	16	MDR
	R	R	S	R	R	R	R	S	R	R	R	R	S	R	
**94**	15	8	23	30	18	21	14	12	24	20	12	24	27	25	MDR
	S	R	S	S	I	S	I	R	S	S	R	S	S	S	
**95**	17	6	21	16	6	15	12	6	23	8	6	20	27	11	MDR
	S	R	S	I	R	R	R	R	S	R	R	I	S	R	
**96**	6	9	20	17	17	20	14	6	24	18	10	23	24	23	MDR
	R	R	S	I	R	I	I	R	S	S	R	S	S	S	
**97**	6	20	18	6	18	6	6	6	18	6	7	6	18	6	MDR
	R	S	S	R	I	R	R	R	S	R	R	R	R	R	
**98**	6	20	21	18	15	21	14	6	6	19	10	24	26	26	MDR
	R	S	S	I	R	S	I	R	R	S	R	S	S	S	
**99**	6	11	22	6	6	6	6	6	18	6	6	6	15	6	MDR
	R	R	S	R	R	R	R	R	S	R	R	R	R	R	
**100**	6	8	21	21	18	22	6	6	6	6	10	23	26	29	MDR
	R	R	S	S	I	S	R	R	R	R	R	S	S	S	
**101**	8	6	16	6	6	17	10	6	10	13	6	19	24	20	MDR
	R	R	I	R	R	R	R	R	R	R	R	R	S	I	
**102**	6	21	21	23	18	24	19	6	25	20	10	23	37	28	MDR
	R	S	S	S	I	S	S	R	S	S	R	S	S	S	
**103**	6	22	23	23	18	22	20	6	25	21	10	25	28	28	MDR
	R	S	S	S	I	S	S	R	S	S	R	S	S	S	
**104**	6	6	22	27	17	23	17	6	6	19	9	24	27	28	MDR
	R	R	S	S	R	S	I	R	R	S	R	S	S	S	
**105**	15	10	21	16	6	15	12	6	23	7	6	19	28	18	MDR
	S	R	S	I	R	R	R	R	S	R	R	R	S	R	
**106**	9	10	20	6	6	10	8	6	6	6	6	6	19	6	MDR
	R	R	S	R	R	R	R	R	R	R	R	R	R	R	

Zone of inhibition in millimeter (mm)

Interpretation: S: Sensitive; I:Intermidate; R: Resistant

TET: Tetracycline; GN: Gentamicin: Amikacin; CIP: Ciprofloxacin; ATM: Aztreonam; PTZ: Piperacillin/tazobactam; AMC: Amoxicillin-clavulanate; SXT:Trimethoprim/sulfamethoxazole; CHL: Chloramphenicol; CXT: Cefoxitin; CRO: Ceftriaxone; MEM: Meropenem; IMP: Imipenem; ETM: Ertapenem

**107**	13	10	19	14	6	15	11	6	21	6	6	17	26	17	MDR
	I	R	S	R	R	R	R	R	S	R	R	R	S	R	
**108**	6	8	24	6	6	6	6	6	16	6	6	6	20	6	MDR
	R	R	S	R	R	R	R	R	I	R	R	R	I	R	
**109**	6	12	24	6	6	6	6	6	17	6	6	7	22	6	MDR
	R	R	S	R	R	R	R	R	I	R	R	R	I	R	
**110**	6	7	18	6	6	6	6	6	17	6	6	6	18	6	MDR
	R	R	S	R	R	R	R	R	I	R	R	R	R	R	
**111**	6	10	23	22	23	24	7	6	23	6	11	26	27	26	MDR
	R	R	S	S	S	S	R	R	S	R	R	S	S	S	
**112**	7	8	20	6	6	17	14	6	6	15	6	26	27	22	MDR
	R	R	S	R	R	R	I	R	R	I	R	S	S	S	
**113**	6	8	21	19	13	22	12	6	6	21	6	25	29	25	MDR
	R	R	S	I	R	S	R	R	R	S	R	S	S	S	
**114**	8	11	22	18	10	15	8	6	6	6	6	17	25	15	MDR
	R	R	S	I	R	R	R	R	R	R	R	R	S	R	
**115**	6	6	20	6	8	7	6	6	6	6	6	6	12	6	MDR
	R	R	S	R	R	R	R	R	R	R	R	R	R	R	
**116**	6	7	21	28	15	24	15	6	6	21	9	25	28	26	MDR
	R	R	S	S	R	S	I	R	R	S	R	S	S	S	
**117**	6	11	23	24	18	23	18	6	25	20	8	24	29	27	MDR
	R	R	S	S	I	S	S	R	S	S	R	S	S	S	
**118**	10	8	20	22	8	19	13	6	24	19	6	23	26	23	MDR
	R	R	S	S	R	I	R	R	S	S	R	S	S	S	
**119**	10	9	20	23	11	19	12	6	23	20	6	24	28	24	MDR
	R	R	S	S	R	I	R	R	S	S	R	S	S	S	
**120**	7	8	17	6	6	14	8	6	6	6	6	13	25	6	MDR
	R	R	S	R	R	R	R	R	R	R	R	R	S	R	
**121**	10	18	22	25	32	23	23	6	11	14	23	30	33	33	MDR
	R	S	S	S	S	S	S	R	R	R	S	S	S	S	
**122**	10	10	18	24	12	18	12	6	22	19	6	23	28	23	MDR

Zone of inhibition in millimeter (mm)

Interpretation: S: Sensitive; I:Intermidate; R: Resistant

TET: Tetracycline; GN: Gentamicin: Amikacin; CIP: Ciprofloxacin; ATM: Aztreonam; PTZ: Piperacillin/tazobactam; AMC: Amoxicillin-clavulanate; SXT:Trimethoprim/sulfamethoxazole; CHL: Chloramphenicol; CXT: Cefoxitin; CRO: Ceftriaxone; MEM: Meropenem; IMP: Imipenem; ETM: Ertapenem

	R	R	S	S	R	I	R	R	S	S	R	S	S	S	
**123**	6	10	20	24	23	22	15	6	6	21	12	23	28	26	MDR
	R	R	S	S	S	S	I	R	R	S	R	S	S	S	
**124**	10	7	22	24	15	20	12	6	23	18	7	24	28	26	MDR
	R	R	S	S	R	I	R	R	S	S	R	S	S	S	
**125**	14	23	25	31	32	27	23	6	26	23	25	28	32	31	Not MDR
	I	S	S	S	S	S	S	R	S	S	S	S	S	S	
**126**	8	13	28	22	14	25	16	6	6	19	13	23	27	24	MDR
	R	I	S	S	I	S	I	R	R	S	R	S	S	S	
**127**	12	10	22	24	18	16	14	6	26	20	6	24	26	27	MDR
	I	R	S	S	I	R	I	R	S	S	R	S	S	S	
**128**	11	8	20	16	15	22	15	6	6	21	8	23	27	25	MDR
	R	R	S	I	R	S	I	R	R	S	R	S	S	S	
**129**	6	13	25	15	6	8	6	6	6	6	6	26	26	17	MDR
	R	I	S	R	R	R	R	R	R	R	R	S	S	R	
**130**	6	11	23	15	15	20	6	6	23	18	8	27	26	24	MDR
	R	R	S	R	R	I	R	R	S	S	R	S	S	S	
**131**	6	8	23	21	16	23	17	6	6	22	8	28	25	23	MDR
	R	R	S	S	R	S	I	R	R	S	R	S	S	S	
**132**	6	10	25	12	6	18	12	6	13	13	6	27	26	25	MDR
	R	R	S	R	R	I	R	R	R	R	R	S	S	S	

Zone of inhibition in millimeter (mm)

Interpretation: S: Sensitive; I:Intermidate; R: Resistant

TET: Tetracycline; GN: Gentamicin: Amikacin; CIP: Ciprofloxacin; ATM: Aztreonam; PTZ: Piperacillin/tazobactam; AMC: Amoxicillin-clavulanate; SXT:Trimethoprim/sulfamethoxazole; CHL: Chloramphenicol; CXT: Cefoxitin; CRO: Ceftriaxone; MEM: Meropenem; IMP: Imipenem; ETM: Ertapenem
