# Supplementary material for: Detection of blaKPC and blaNDM carbapenemase genes among Klebsiella pneumoniae isolates in Addis Ababa, Ethiopia: Dominance of blaNDM
Source: PLoS One. 2022 Apr 27;17(4):e0267657. doi: 10.1371/journal.pone.0267657 (PMC9045624; doi:10.1371/journal.pone.0267657)
Supplement: S2 Table — mCIM: modified Carbapenem Inactivation Method, ESBL: Extended-spectrum β-lactamase, Interp: Interpretation, ZI in mm: Zone of inhibition in millimeter, M: Male, F: Female, CSF: Cerebrospinal fluid, ICU: Intensive Care Unit. (PDF) [file pone.0267657.s002.pdf]

S2 Table. Patient characteristics, mCIM test result, ESBL result and carbapenemase genes of 39 carbapenem non-susceptible *K. pneumoniae* isolates at Tikur Anbessa Specialized Hospital

Code	Gene	mCIM test result		ESBL with combined disc test	Sex of patient	Age of patient in years	Source of isolation	Hospital unit
		ZI in mm	Interp.					
**14**	bla_{NDM}	6	Positive	Negative	M	13	Urine	ICU
**16**	bla_{NDM}	10	Positive	Negative	F	26	Urine	Medical ward
**20**	Negative	22	Negative	Positive	F	6	Urine	Pediatrics ward
**27**	Negative	23	Negative	Positive	F	60	CSF	ICU
**31**	bla_{NDM}	6	Positive	Negative	M	1	Wound	ICU
**32**	bla_{NDM}	12	Positive	Negative	M	5	Sputum	Pediatrics ward
**33**	bla_{NDM}	6	Positive	Negative	F	5	Urine	ICU
**39**	Negative	20	Negative	Positive	F	<1	Blood	Pediatrics ward
**49**	bla_{NDM} & bla_{KPC}	6	Positive	Positive	M	60	CSF	ICU
**50**	bla_{NDM}	6	Positive	Negative	M	4	Urine	Pediatrics ward
**51**	bla_{NDM}	6	Positive	Negative	M	3	Blood	Pediatrics ward
**54**	bla_{NDM}	6	Positive	Negative	M	6	CSF	Pediatrics ward
**56**	Negative	20	Negative	Negative	F	12	Urine	ICU
**57**	bla_{NDM}	6	Positive	Negative	F	5	Urine	Pediatrics ward
**61**	bla_{NDM}	10	Positive	Negative	M	38	Blood	Medical ward
**63**	Negative	11	Positive	Negative	M	50	Urine	Medical ward
**64**	bla_{NDM}	6	Positive	Negative	M	< 1	Blood	Pediatrics ward
**67**	bla_{NDM}	14	Positive	Negative	M	30	Blood	Medical ward
**70**	Negative	19	Negative	Positive	F	4	Blood	Pediatrics ward
**74**	Negative	12	Positive	Negative	M	41	Wound	Surgical ward
**81**	bla_{NDM}	6	Positive	Negative	F	< 1	Wound	Pediatrics ward
**83**	bla_{NDM}	6	Positive	Positive	F	28	Blood	Surgical ward
**87**	bla_{NDM}	11	Positive	Negative	M	17	Wound	Outpatient
**90**	bla_{NDM}	8	Positive	Negative	M	< 1	Urine	Pediatrics ward
**93**	bla_{NDM}	6	Positive	Positive	F	29	CSF	Surgical ward
**95**	Negative	21	Negative	Positive	M	<1	Blood	ICU
**97**	bla_{NDM}	6	Positive	Negative	F	23	Urine	ICU
**99**	bla_{NDM}	6	Positive	Negative	M	62	CSF	ICU
**101**	bla_{NDM}	6	Positive	Positive	F	32	Wound	Surgical ward
**105**	Negative	22	Negative	Positive	M	<1	Blood	ICU
**106**	Negative	20	Negative	Negative	M	2	Blood	Pediatrics ward
**107**	Negative	20	Negative	Positive	F	5	Wound	Pediatrics ward
**108**	bla_{NDM}	6	Positive	Positive	F	26	Urine	Medical ward
**109**	bla_{NDM}	12	Positive	Negative	M	5	Urine	Pediatrics ward
**110**	bla_{NDM}	6	Positive	Negative	M	11	Wound	ICU
**114**	bla_{NDM}	6	Positive	Positive	M	2	Blood	ICU
**115**	Negative	21	Negative	Negative	M	<1	Blood	ICU
**120**	bla_{NDM}	13	Positive	Negative	M	< 1	Blood	Pediatrics ward
**129**	Negative	23	Negative	Positive	F	5	Urine	Pediatrics ward

mCIM: modified Carbapenem Inactivation Method; ESBL: Extended spectrum β-lactamase; Interp: Interpretation;
ZI in mm: Zone of inhibition in millimeter; M: Male; F: Female; CSF: Cerebrospinal fluid; ICU: Intensive Care Unit
