# Supplementary figures and images for: Detection of blaKPC and blaNDM carbapenemase genes among Klebsiella pneumoniae isolates in Addis Ababa, Ethiopia: Dominance of blaNDM
Source: PLoS One. 2022 Apr 27;17(4):e0267657. doi: 10.1371/journal.pone.0267657 (PMC9045624; doi:10.1371/journal.pone.0267657)

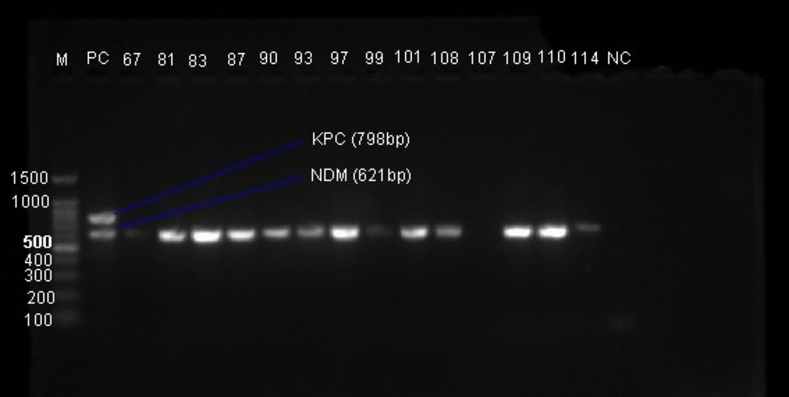

Supplement: S1 Fig — Lane M: 100bp DNA ladder; PC: Positive control, Lanes 67–114: K. pneumoniae isolates, NC: Negative control. (TIF) [file pone.0267657.s003.tif]

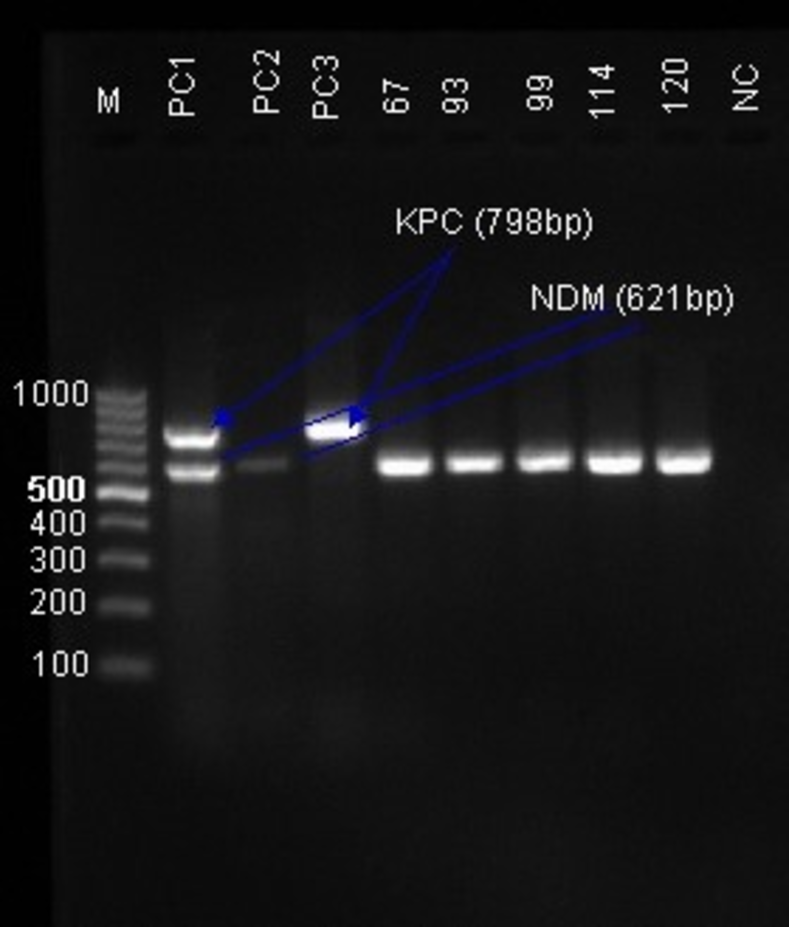

Supplement: S2 Fig — Lane M: 100bp DNA ladder, PC1, PC2 & PC3: Positive control, Lanes 67–120: K. pneumoniae isolates, NC: Negative control. (TIF) [file pone.0267657.s004.tif]
